# Measurement of Pneumatic Valve Flow Parameters on the Test Bench with Interchangeable Venturi Tubes and Their Practical Use

**DOI:** 10.3390/s23136042

**Published:** 2023-06-29

**Authors:** Ryszard Dindorf

**Affiliations:** Faculty of Mechatronics and Mechanical Engineering, Kielce University of Technology, Al. Tysiaclecia Panstwa Polskiego 7, 25-314 Kielce, Poland; dindorf@tu.kielce.pl

**Keywords:** flow parameters, pneumatic valves of different sizes, flow test bench, automated flow measurement, interchangeable venturi tubes

## Abstract

A test bench with interchangeable venturi tubes was built to automatically measure the flow parameters of pneumatic valves of a wide range of sizes. This measuring stand contained components recommended by the ISO 6358 standard, an individually configured flow meter circuit, and HMI measurement and control panels. The flow meter circuit, individually configured with interchangeable venturi tubes, bypass loops, and Setaram thermal microflow meter, was calibrated using Molbloc/Molbox equipment. The tuning curve and theoretical flow rate characteristics of the tested valve were fitted to the flow rate measurement data. The best fit value of the critical pressure ratio was obtained using the numerical method of least squares minimization. The pneumatic valve with measured flow parameters was compared with data from the catalogue on the discharge characteristics of the compressed air tank. A practical solution for high-pressure tank discharge time using two valves connected in series to the hybrid tricycle bike (HTB) pneumatic propulsion system is presented. This article presents a solution to the practical problem of measuring the flow parameters of industrial pneumatic valves with a wide range of nominal diameters on a test bench with replaceable venturi tubes.

## 1. Introduction

Pneumatic valve measurements are used to determine their characteristics and flow parameters, which are then used in the control, diagnostics, monitoring, and safety of pneumatic systems. Pneumatic systems use high- and low-pressure compressed air to process, transfer, and store energy. High-pressure pneumatics are used in production processes (300 bar), drive systems (200 bar), and energy storage tanks (500 bar). In [1], a computational simulation was performed and the experimental work on fluid flow was presented through a high-pressure pneumatic circuit used in a blowing machine. Low-pressure pneumatics, operating at a pressure of up to 10 bar, are widely used in manufacturing, mining, energy, maritime, rail and road transport, drive systems, and medical equipment, as well as in the technological processes of conveying, blowing, shaping, cleaning, and cooling [2]. Pneumatic technologies and products play a vital role in a wide variety of manufacturing systems, providing reliable performance for a wide range of actuation and movement tasks. Pneumatic systems consist of compressors, compressed air plants, compressed air tanks, and compressed air consumers [3]. Pneumatic systems can be used for various purposes: technological processes, drives and controls, energy storage, braking systems, and others. The efficient operation of a pneumatic system depends on the performance of the control and measurement processes, in which the pneumatic control valves play a key role. Properly selected pneumatic control valves ensure the effectiveness and performance of the technological process, provide measurable savings, and reduce the operating costs of the pneumatic system. The size and flow characteristics of the valves must meet the requirements of pneumatic systems [4]. When sizing a control valve, the rule of thumb is to size it so that it operates somewhere between 20 and 80% open at the maximum required flow rate and, whenever possible, not much less than 20% open at the minimum required flow rate. To properly select a control valve, the following parameters must be known: medium, pressures, drop pressure, flow rate, and temperature. The maximum flow rate is used to select the valve size, the minimum flow rate to check the turndown requirement, and the normal flow rate to check the valve control. Pneumatic valves are devices used to regulate the flow and pressure of compressed air in a pneumatic system. Pneumatic valves can be used for various applications, such as shutting down, switching, throttling, or adjusting the direction of air flow. Pneumatic control valves must be precisely adapted to the specific application of pneumatic systems. Properly selected pneumatic control valves last longer than mismatched or incorrectly sized valves. The procedure to select the size of pneumatic valves consists of matching their characteristics and flow parameters to the flow dynamics in pneumatic systems [5,6]. The effective use of a pneumatic valve should include an analysis of air flow efficiency and pressure loss and cost reduction. There are many types of valves available, with some advantages and restrictions. The basic requirements and the selection of valve size depend on their ability to perform specific functions, such as the ability to throttle or control the direction of flow rate, no turbulence and low flow resistance, quick opening and closing, fast response, tight closure, or opening at a given pressure. Pneumatic valves are individually selected to meet the specific requirements for the controlled, safe, flexible, and economical operation of pneumatic systems. Pneumatic valves are sized on the basis of their operating parameters, such as temperature, pressure, nominal flow, and nominal diameter. The operating pressure is the normal pressure in the range of 0.4 to 0.7 MPa, at which the pneumatic valves operate. The nominal flow rate is a measure of the volumetric flow that passes through the valve under specified nominal operating conditions. The nominal flow rate is the flow volume (under normal conditions) that passes through a valve with an upstream pressure *p*_1_ = 6 bar (7 bar absolute pressure) and a pressure drop of 1 bar, corresponding to a downstream relative pressure *p*_2_ of 5 bar (6 bar absolute pressure). The nominal diameter DN (fr. diamètre nominal) is a standard indicator that is used as a reference to evaluate valve size. The nominal diameter of the valve matches the nominal diameter of the connected pipe. The match of the nominal diameters of the valve and the pipeline avoids oversizing the valve, which could lead to the unstable operation of the pneumatic systems. Additionally, if the valve is undersized, it can cause a high-pressure drop and damage the pneumatic components.

The data sheets of industrial valves include flow coefficients *C*v in imperial units gpm according to the ANSI(NEPA) T3.21.3: 1990 standard [7] or *K*v in metric units m^3^/h (or *k*v in L/min) according to the VDI/VDE 2173: 2022 standard [8]. The relationship between *C*v and *K*v is expressed as follows, *C*v = 1.16 *K*v and *K*v = 0.862 *C*v. When applying the flow factors *C*v or *K*v to pneumatic valves, various complications occur as they are determined using water as a test fluid. ANSI/(NFPA)T3.21.3-1990 will provide a common reference for test methods to be used on pneumatic elements. The VDI/VDE 2173: 2022 standard summarises the essential requirements of the existing standards and makes them available for practical application. It applies to all types of industrial process control valves. The procedures for sizing industrial process control valves are based on accepted mathematical methods, such as those specified in the ANSI/ISA-75.01.01: 2012 standard [9]. ANSI/ISA-75.01.01-2012 includes equations for predicting the flow of compressible and incompressible fluids through control valves. The equations for incompressible flow are based on standard hydrodynamic equations for Newtonian incompressible fluids. They are not intended for use when non-Newtonian fluids, fluid mixtures, slurries, or liquid–solid conveyance systems are encountered. The equations for incompressible flow may be used with caution for non-vaporizing multi-component liquid mixtures. To calculate the air flow through the pneumatic valve, it is necessary to use a conversion of these factors. Using this method to calculate the air flow rate will not provide accurate results. A particular problem is the small pressure drop across the pneumatic valve, which in the valve seat is 15% of the inlet pressure. From an economic and environmental point of view, it is justified to use two flow parameters, such as sound conductance *C* and critical pressure ratio *b* recommended by the ISO 6358: 2013 standard [10]. The ISO 6358-1:2013 standard specifies requirements for the installation of the test, the test procedure, and the presentation of the results for the steady state method. Value *C* is the maximum valve flow rate when the air passing through reaches the sonic flow condition (choked flow). Value *b* is the critical pressure ratio between the downstream pressure and the upstream pressure when the flow condition changes from subsonic to sonic or vice versa. In paper [11], algorithms are presented to calculate the nominal flow rate *q*_vn_ and the flow factor *K*v as a function of the volume flow sonic conductance *C* and the critical pressure ratio *b*. In this paper, two methods are presented that were used to determine the sonic conductance *C* and the critical pressure ratio *b*, when the flow coefficient *K*_v_ or the nominal flow rate *q*_Nnom_ are known. These two approaches improve the selection of flow parameters for pneumatic control valves. The alternative flow parameter is the effective sectional area *S* in mm^2^ according to the JIS B 8390: 2016 standard [12]. JIS B 8390: 2016 deals with the determination of the flow rate characteristics of components using compressible fluids. This standard is an important tool in determining the flow rate expressed by the effective area S. The *S* represents the maximum constant flow in choked flow. It also expresses the size of the valve in direct relation to the speed of the actuator. The conversion of the sonic conductance *C* in dm^3^ s^−1^∙bar^−1^ to the effective sectional area *S* in mm^2^ applies: *S* = 5 *C*. The flow parameters *C* and *b* contained in the data sheets often differ from their actual values, which were confirmed by the experimental determination of the characteristic of the pneumatic control valve [13]. The object of this article is to test the properties of pneumatic elements. These elements are part of the equipment to measure the parameters of rotary air motors. A comparison of the measured data with the mathematical model revealed minor variations. The flow parameters *C* and *b* of the pneumatic valves are not always given in the manufacturer’s data sheets. Some pneumatic components manufacturers do not measure valve flow parameters, but only provide their nominal DN diameters in the data sheets.

## 2. Measurement Methods to Determine the Flow Parameters of Pneumatic Components

**Figure 1:**
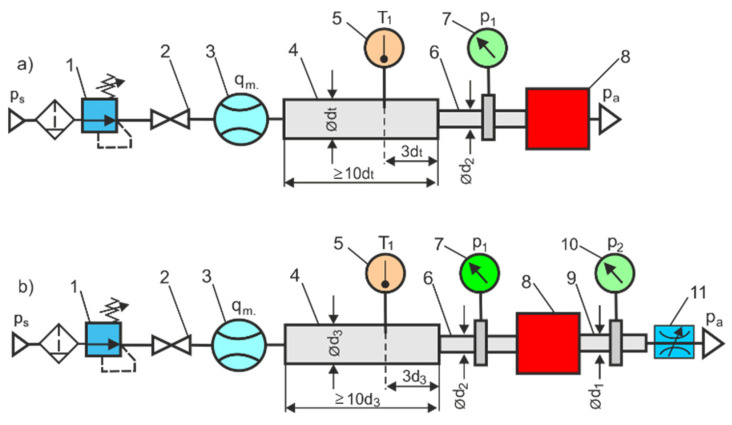
A test set-up according to the ISO 6358 standard: (**a**) circuit for the exhaust-to-atmosphere test, (**b**) circuit for the in-line test, 1—adjustable pressure reducing valve, 2—shut-off valve, 3—mass flow meter, 4—temperature measuring tube, 5—temperature measuring instrument, 6—upsteram pressure measuring tube, 7—upstream pressure gauge, 8—pneumatic component under test, 9—downstream pressure measuring tube, 10—downstream pressure gauge, 11—flow control valve [9].

Direct or indirect measurement methods are used to measure the characteristics and flow parameters *C* and *b* of pneumatic components (valves, nozzles, orifices, venturi tubes, etc.). The test methods used to directly measure the flow characteristics of pneumatic components are based on the ISO 6358 standard, which has been validated for an ideal gas mass flow model in low-pressure systems up to 1.6 MPa. Since flow measurements are actually made for real gas (humidity air is treated as real gas), they can be subject to error. This method of measurement can also be uncertain at higher pressures. The direct measurement method according to ISO 6358 has many stringent requirements for the testing equipment and the accuracy of the measurement instrumentation. The basic test circuits according to the ISO 6358 standard, the circuit for the in-line test and the circuit for the exhaust-to-atmosphere test, that are used to directly determine the flow parameters of pneumatic components, are shown in Figure 1.

The measurement methodology according to ISO 6358 is based on the manual set of upstream pressure using an adjustable pressure regulator (reducing a valve), which makes the measurement process long and inaccurate and subject to human error. The main measurement problem is that the pressure regulator is not directly upstream of the valve under test. The measurement is carried out at a constant upstream pressure before the tested valve. In an in-line test circuit, the pressure drop between the pressure regulator and the pneumatic valve under test is adjusted by the flow control valve.

The ISO 6358 standard test is widely used to measure pneumatic valve flow parameters. In the study [14], a method was proposed to calculate the sonic conductance of a short tube orifice. First, the formula to calculate the sonic conductance was derived from the continuity equation, the momentum equation, and the definition of the flow characteristic. The flow characteristics of the individual outlets were then measured using the ISO 6358 upstream constant pressure test method. The critical pressure ratio has little effect on the sonic conductance of a short tube orifice, and it can be set to 0.5 when calculating the sonic conductance in engineering applications. The formula proposed in this study is very accurate with a mean error of 3%. In the paper [15], the mass flow model according to ISO 6358 was used to measure the parameters *C* and *b* for real gas in a high-pressure range of up to 30 MPa. This publication recommends an easy-to-use model to calculate the mass flow of real gases. For this purpose, the existing mass flow model, based on equations for the ideal gas range according to ISO 6358, is applied and verified for real gases. The critical conductance parameters *C* and the critical pressure ratio b are obtained, applied, and checked in the real gas state space. The mass flow models for gaseous media describe the relationship between gas flow through throttle elements depending on pressure, temperature*,* and type of medium. These models are used to calculate pneumatic components, simulate pneumatic systems*,* or plan facilities. Article [16] proposes a test bench to measure flow rates according to the ISO 6358 standards, allowing partially automated testing, convenient installation, good configuration, ease of use, and measurement precision. The measurement station was realised in such a way as to obtain versatility of use, with a wide range of components that are different in size and flow characteristics, the automation of the acquisition and calculation processes, rapidity to execute a complete test campaign thanks to a closed-loop regulation of upstream pressure, data quality control and one-button acquisition, high accuracy, ease of use, and user friendliness by providing all the numerical and graphical information the operator needs to keep under control. In the work [17], the results of the mass flow rate measurements obtained using the direct method according to the ISO 6358 standard and the indirect method during the unload (discharge) of an air tank were compared. The indirect method uses the characteristic tank discharge time method and assumes a measurement cost lower than the ISO 6358 standard procedure. The sonic conductance obtained from the characteristic discharging time method is less than that obtained from the ISO 6358 standard. In this measurement method, the test stand is not large and the accurate measurement of discharge parameters such as pressure, average temperature, or specific volume are also not required.

There are a number of methods for measuring air flow parameters, each with its own advantages and disadvantages in terms of gas type, measurement range, and accuracy. In ISO standards, orifice plates, nozzles, venturi tubes, and venturi nozzles are used to measure air flow. Designing measurement stations according to ISO standards is beneficial due to the certification of test methods, the accuracy of assessment measurements, and worldwide recognition.

**Figure 2:**
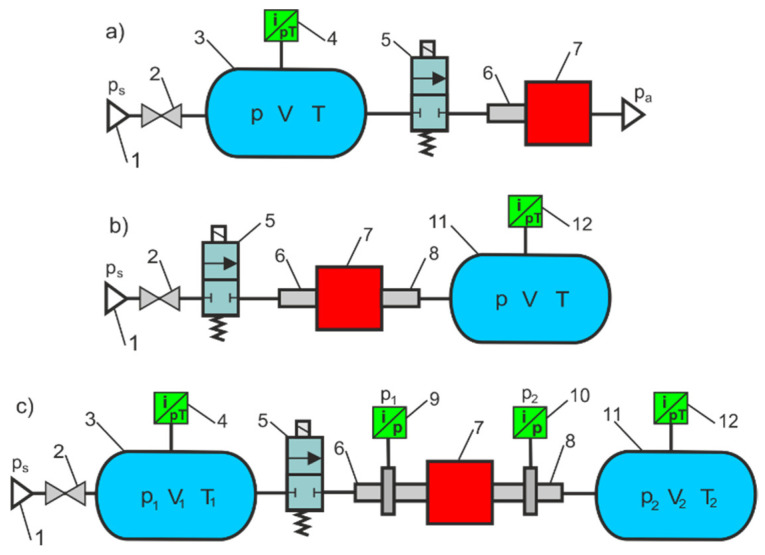
The test bench for indirect measurement of the flow parameters of pneumatic components: (**a**) discharge test, (**b**) charge test, (**c**) tank-to-tank test: 1—compressor air source, 2—shut-off valve, 3—supply tank, 4—dual pressure and temperature transducer, 5—switching valve, 6—upstream pressure flow measurement tube, 7—pneumatic component under test, 8—downstream pressure measurement tube, 9—upstream pressure and temperature transducer, 10—downstream pressure transducer, 11—exhaust tank, 12—dual pressure and temperature transducer.

Indirect flow measurements are usually less expensive and less complicated than direct flow measurements. Indirect flow measurements do not require the use of mass flow meters or high-efficiency compressors. The following tests are used for indirect flow measurements: simple discharge test, vacuum charge test, isothermal discharge test, charge test, discharge test, and tank-to-tank test. The schematic diagrams of the test bench for the indirect measurement of the flow characteristics and parameters of the pneumatic components are shown in Figure 2.

In [18], a new indirect method was developed to measure flow in a pneumatic pipeline system. This method allows the measurement of a controlled leak in the CAS branch using a portable measuring device (PMD). Based on the patent of the authors, an automatic measurement system (AMS) was developed to measure the leakage flow rate in industrial CAS. The article [19] describes a method for measuring the characteristics of the flow rate of a proportional pressure regulator based on the charge and discharge of the tank through the valve while monitoring the downstream pressure, the upstream pressure, the temperature of the supply air, and the pressure inside the tank. In the work [20], a new discharge method was proposed for the flow characteristics of high-pressure pneumatic servo valves, based on the principle of sonic discharges of series connection orifices. In the study [21], a novel discharge method was used to identify the flow rate characteristics of a pneumatic valve using instantaneous polytropic exponents.

## 3. Test Bench with Individually Configured Flow Meter Circuits

**Figure 3:**
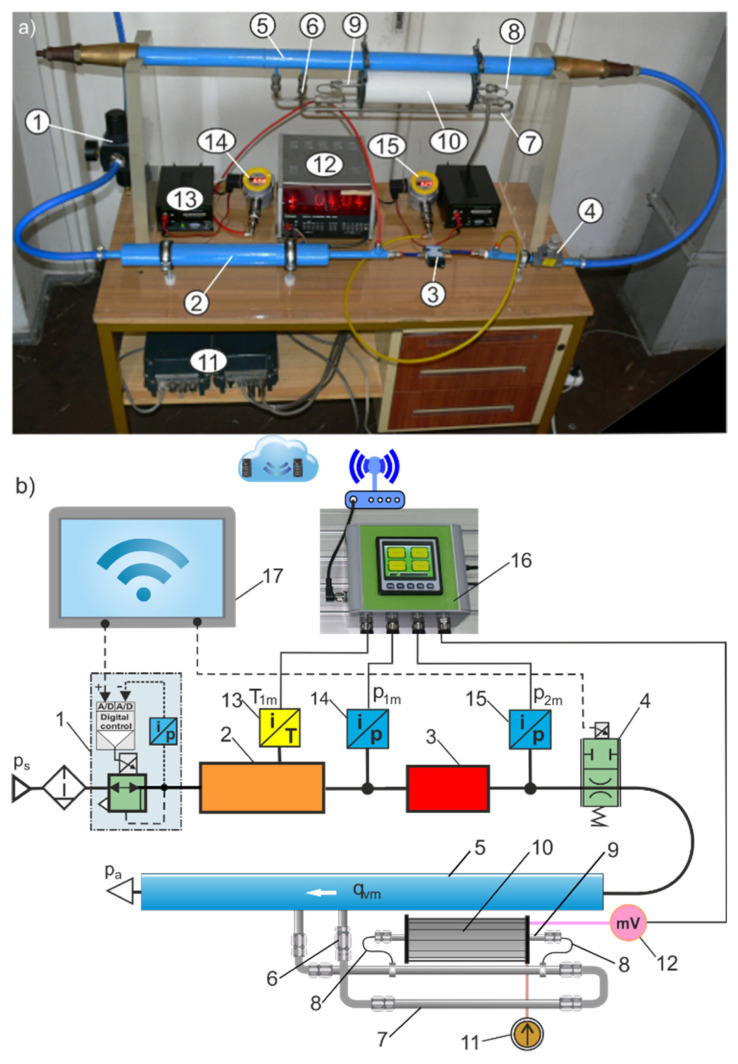
View (**a**) and diagram (**b**) of the measuring test bench with interchangeable venturi tubes: 1—digitally controlled proportional pressure regulator, 2—upstream measuring tube, 3—valve under test, 4—proportional flow control valve, 5—interchangeable venturi tube, 6—calibrated orifice, 7—main bypass loop, 8—meter bypass loop with capillary tubes, 9—heated tube, 10—Setaram thermal microflow meter, 11—power supply, 12—millivolt meter, 13—temperature meter, 14, 15—digital pressure sensors, 16—HMI measurement panel, and 17—HMI control panel.

The thermal flow meter for the industrial measurement of compressed air has specific flow rate measurement ranges that limit their use to a wide range of valve sizes. Examples of the nominal flow rates of industrial pneumatic valves: flow control valve *q*_v*N*_ = 20–200 L/min (1.2–12 m^3^/h), proportional directional control valve *q*_v*N*_ = 100–2000 L/min (6–120 m^3^/h), and directional valve *q*_v*N*_ = 500–4200 L/min (30–252 m^3^/h). The purchase of several expensive thermal flow meters adapted to different flow rates of pneumatic valves usually exceeds the financial capabilities of research laboratories. Therefore, a measuring stand was built to measure the flow characteristics and parameters of pneumatic valves tested with a wide range of sizes, from very small to very large nominal flows. A wide range of flow rate measurements are possible by adopting replaceable Venturi tubes. The measuring stand contains components recommended in the ISO 6358 standard for the online testing circuit. The measuring stand has also been equipped with a flow meter circuit and HMI measurement and control panels. The flow meter circuit contains interchangeable venturi tubes, a Setaram thermal microflow meter (called Setaram) [22], and two bypass loops, main and side. The main bypass loop contains a calibrated orifice. The Setaram is placed in the side bypass loop. The heated tube of the Setaram in the side bypass loop is connected using capillary tubes to the main bypass loop. The view and diagram of the measuring test bench with replaceable venturi tubes for the automatic measurement of pneumatic valve flow rates in a wide range of sizes are shown in Figure 3.

The flow meter circuit has the following properties:

- The interchangeable venturi tubes can be adapted to the flow measurement range of the pneumatic valves.
- Pressure losses in bypass loops depend on the choice of calibrated orifice and pipe dimensions.
- The flow measurement is independent of the gas pressure and temperature, allowing for the direct measurement of the volumetric flow rate *q*_v_ in m^3^/h.
- Interchangeable venturi tubes in the nominal diameter range from 0.015 to 0.080 m significantly extend the flow rate measurement range from 2.5 to 250 m^3^/h for a given upstream pressure.
- The upstream pressure can have a different range, up to 1, 2, and 5 MPa, depending on the dimensions of the bypass loops.

**Figure 4:**
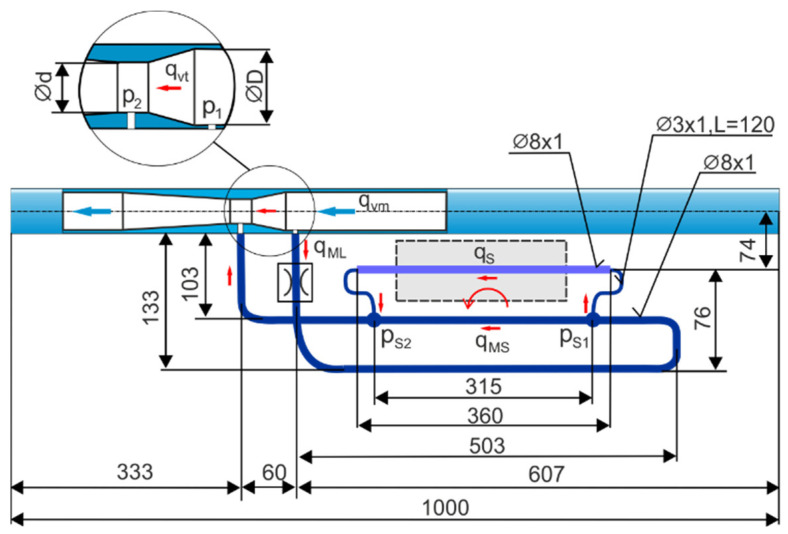
Dimensions of the flow meter circuit with interchangeable venturi tubes, the main bypass loop, and the side bypass loop.

Figure 4 shows the dimensions of the flow meter circuit with interchangeable venturi tubes, the main bypass loop, and the side bypass loop to measure the flow rate in a pressure range of up to 1 MPa.

The interchangeable, classically machined venturi tubes for gas, have been selected for the flow meter circuit. Table 1 lists the nominal diameter *D* and diameter ratio *β* of the venturi tubes adapted for different flow rates at pressures of up to 1 MPa.

Placing the Setaram thermal microflow meter in the side bypass loop enables the measurement of the flow rate from very low (up to several dm^3^/h) to high (up to 250 m^3^/h) at pressures of up to 1 MPa. For the parallel connection of the side loop, the main loop, and the venturi tube, the following equation for volumetric flow rates was written.

(1) $$q_{\mathrm{vm}}=q_{vt}+q_{ML}=q_{vt}+q_{MS}+q_{S}$$

then

(2) $$\frac{q_{\mathrm{vm}}}{q_{S}}=1+\frac{q_{MS}}{q_{S}}+\frac{q_{vt}}{q_{S}}$$

where *q*_v*m*_ is the measured flow rate, *q_S_* is the flow rate in the Setaram heated tube, *q*_v*t*_ is the flow rate through the venturi tube, and *q_MS_* is the flow rate in the main bypass loop tube section.

For the side bypass loop under laminar flow conditions, the pressure loss is as follows,

(3) $${\Delta p}_{S}=p_{S1}-p_{S2}=R_{S}\;q_{S}=R_{MS}\;q_{MS}$$

Then (2) takes the form:

(4) $$\frac{q_{\mathrm{vm}}}{q_{S}}=1+\frac{R_{S}}{R_{MS}}+\frac{q_{vt}}{q_{S}}=K+\frac{q_{vt}}{q_{S}}\;\Rightarrow \;q_{\mathrm{vm}}=q_{S}\left(K+\frac{q_{vt}}{q_{S}}\right)$$

where *R_S_* is the flow resistance in the side bypass loop, *R_SM_* is the flow resistance in the main bypass loop tube section, and *K* is the calibration constant

(5) $$K=1+\frac{R_{S}}{R_{MS}}$$

The theoretical volume flow rate through the venturi tube for a compressible fluid (gas) is calculated using the following formula [23],

(6) $$q_{vt}=\frac{q_{m}}{\rho_{m}}=\frac{C_{d}}{\sqrt{1-\beta^{4}}}\;\varepsilon \;\frac{\pi }{4}\;d^{2}\sqrt{\frac{2\;\Delta p}{\rho_{a}}}$$

where *q_m_* is the mass flow rate through the venturi tube in kg/s, *q*_v*t*_ is the volume flow rate through the venturi tube in m^3^/s, *C_d_* is the discharge coefficient, *C_d_ =* 0.987, *ε* is the expansibility factor, *ε* = 0.978, *β* is the diameter ratio, *β* = *d*/*D*, *d* is the diameter of the throat in m, *D* is the nominal diameter of the tube in m, Δ*p* is upstream-to-throat differential pressure in Pa, Δ*p* = *p*_1_ − *p*_2_, *p*_1_ is the static upstream pressure in Pa, *p*_2_ is the static pressure in the throat in Pa, *ρ_m_* is the air density in kg/m^3^ under measurement conditions, for the measured pressure *p*_2*m*_ and temperature *T*_1*m*_, *ρ_m_* = *p*_2*m*_ *R*^−1^*T*_1*m*_^−1^, and *R* is the individual gas constant of air in J kg^−1^ K^−1^.

**Figure 5:**
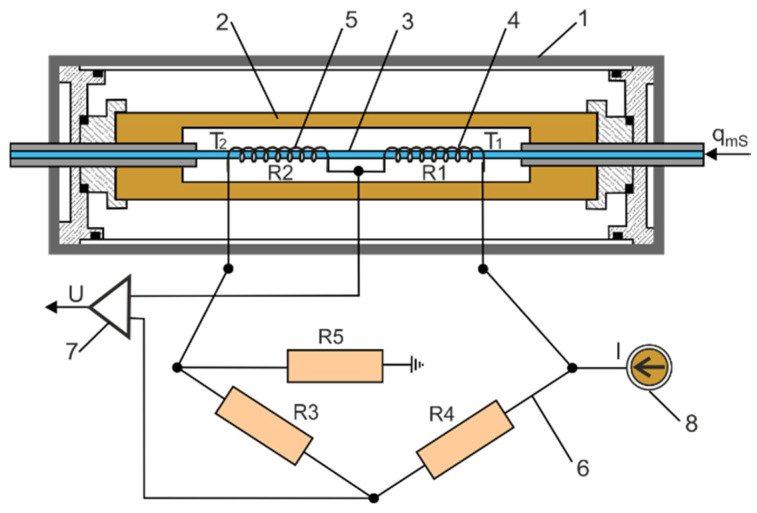
Setaram thermal microflow meter with electrical resistance bridge: 1—sealed cylindrical container, 2—thermal shield, 3—heated tube, 4—resistance winding on the inlet side, 5—resistance winding on the outlet side, 6—resistance bridge, 7—amplifier, 8—constant-current power supply.

The Setaram thermal microflow meter is placed inside a sealed cylindrical container with a diameter of ∅80 mm and a length of 262 mm. On the axis of this cylinder, there is a thin-walled heated metal tube with an outer diameter of ∅8 mm, stiffened and sealed at the ends, through which compressed air flows. The heated tube and the resistance windings are surrounded by a thermal shield. Two resistance windings with a high-temperature coefficient of resistance (TCR) are wound on the outside of this tube. The resistance windings are arranged symmetrically on the inlet and outlet sides of the heated tube. The stabilized electric current flowing through the resistance windings heats the metal tube. The Setaram thermal microflow meter with electrical resistance bridge arrangement is shown in Figure 5. The metal tube on a Setaram microflow meter is heated in constant power (CP) mode. When the flow meter operates in CP mode, the winding voltage is controlled so that the output power is constant [24]. The volume flow rate *q_S_* in the heated Setaram tube is inversely proportional to the temperature difference Δ*T* = *T*_2_ − *T*_1_, as follows [25],

(7) $$q_{S}=\frac{f_{S}\;\left(P-L\right)}{\rho_{a}\;c_{p}\;\left(T_{2}-T_{1}\right)}$$

where *q_S_* is the volume flow rate of the gas in the Setaram heated tube in m^3^/s, *f_S_* is the proportionality factor of the Setaram, *P* is the input power converted into heat energy in W, *L* is the conduction loss in W, *c_p_* is the specific heat of the gas at constant pressure in J kg^−1^ K^−1^, *T*_1_ is the temperature of the gas (tube wall) before the heater in K, and *T*_2_ is the temperature of the gas (tube wall) behind the heater in K.

The resistances array creates a Wheatstone bridge that is unbalanced during airflow due to the temperature difference of the resistance windings at the inlet and outlet of the tube. There is no airflow, the bridge is balanced, and the output is zero. The Setaram has a large time constant and requires a long warm-up time. Before measurements are taken, the thermal flow meter must be turned on for 120 to 180 min to stabilize the temperature. The output voltage signal is directly proportional to the difference in average temperature in the resistance windings. Based on the resistance bridge in Figure 5, the output voltage of the bridge was determined,

(8) $$U=\frac{R_{0}R_{3}\;\alpha \;\left({\bar{T}}_{2-}{\bar{T}}_{1}\right)}{\left[2+\alpha \;\left(T_{1}+T_{2}\right)\right]R_{0}+2\;R_{3}}\;I$$

where *U* is the voltage in V, *I* is the current in A, *α* is the thermal coefficient of resistance in 1/K, *R*_0_ is the resistance of the windings at ambient temperature (*T_a_* = 293.15 K) in Ω, *R*_3_ is the constant resistance in Ω, ${\bar{T}}_{1}$, ${\bar{T}}_{2}$ are the average temperatures of the resistance winding in K.

The output electrical signal (voltage) is used to record the measurement data and control the measurement cycle. Setaram has current adjustment, as well as zero-point, linearity, and sensitivity settings.

Thanks to interchangeable venturi tubes, it is possible to measure flow rates through pneumatic valves with a nominal flow of 2.5 to 250 m^3^/h. On a calibrated measuring circuit with interchangeable venturi tubes of a specific nominal size, the flow parameters of the pneumatic valves are measured in the same range of nominal flow. In industrial pneumatics, pneumatic valves with a wide range of sizes are used, for which it is difficult to determine the flow parameters using theoretical calculations. The flow parameters of pneumatic valves have an important effect on the performance of pneumatic control systems. The correct selection of pneumatic valves depends on the type of medium, working pressure, pressure drop, flow rate, and temperature. Determining the flow rate is significant because the maximum flow rate is used to select the valve size, the minimum flow rate is used to check the turndown requirement, and the normal flow rate is used to check the valve control.

## 4. Calibration of the Individually Configured Flow Meter Circuit

**Figure 6:**
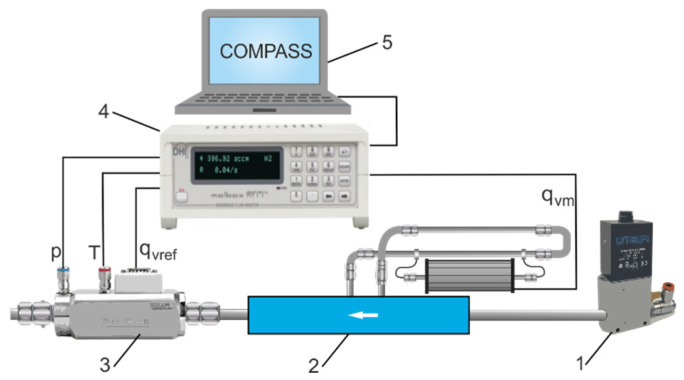
Calibration test bench for the flow meter circuit: 1—pressure regulator, 2—calibrated flow meter circuit, 3—Molbloc-S, 4—Molbox1+, 5—PC with Compass calibration software.

The Setaram principle of measurement implies that the power needed to maintain the temperature difference is directly proportional to the mass flow rate. This relationship between power and mass flow rate is established during calibration. Each thermal flow meter has slightly different heat transfer properties. Therefore, the flow meter circuit, individually configured with interchangeable venturi tubes, bypass loops, and the Setaram thermal microflow meter, must be calibrated. Molbloc/Molbox equipment was used to calibrate the flow meter circuit as shown in Figure 4. The Molbox is the flow terminal from Fluke Calibration. Molblocs are calibration flow elements that are connected in series with the flowmeter to be calibrated. Calibration laminar or sound elements are used. The COMPASS Molbox software is used for flow meter calibration. The Molbloc/Molbox system has an operational range of less than 1 sccm (standard cubic centimetre) for the laminar calibration to more than 5000 slm (standard litre per minute) for the sonic calibration. A Molbloc/Molbox system can be used to calibrate the volume flow meter if the actual gas pressure and temperature conditions at the flow meter location are known. Many very common flow meters are based on volume flow measurement principles and are calibrated in volume flow units for mass flow. If calibration is performed under conditions other than the flow meter’s measurement conditions, a correction is necessary to properly correlate the output flow rate of the calibrated flow meter with the Molbox reference flow. The calibration of the individually configured flow meter circuit was performed in an accredited flow meter laboratory certified by the Central Office of Measures. The calibration of the flow meters is carried out in a pressure range of up to 1.2 MPa, a temperature of up to 331.15 K, and with an accuracy of ±0.2% RD (percentage of reading). The calibration consisted of a direct comparison between the flow rate measurements *q*_v*m*_ in the calibrated flow meter circuit and the reference flow rate *q*_v*ref*_ of the Molbloc flow meter. The diagram of the calibration test bench for the calibration of the flow meter circuit with the Molbloc/Molbox system is shown in Figure 6.

**Figure 7:**
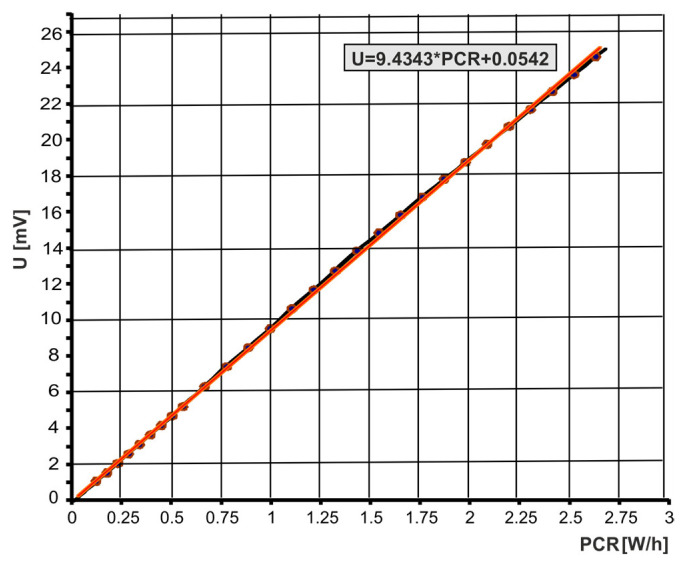
Calibration curve *U* = *f*(*PCR*) of the flow meter.

**Figure 8:**
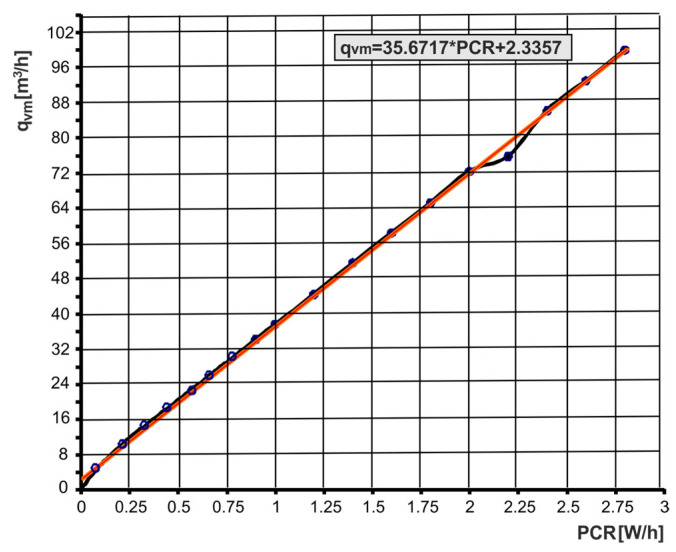
Calibration curve *q*_v*m*_ = *f*(PCR) of the flow meter.

The calibration of the individually configured flow meter circuit consisted of determining the calibration curves of the voltage *U* in mV and the volume flow rate *q*_v*m*_ in m^3^/h for the power change rate (PCR) in W/h (units that indicate the change in power over time). The power change rate (PCR) at W/h is the calibration parameter of the Setaram microflow meter. The calibration curves were linearly approximated. The linearization of the calibration curves increases the accuracy and repeatability of the flow rate measurement and is also essential in automating this measurement. For the purposes of determining the characteristics and flow parameters of pneumatic valves, the flow meter circuit with an interchangeable venturi tube with a nominal diameter of *D* = 0.065 m was calibrated. The calibration curves performed are shown in Figure 7 and Figure 8. Calibrated flow meter circuits have an accuracy of 1.5% and a repeatability of 0.5% full-scale (FS). From the calibration curve *U* = *f*(PCR) the PCR value is read, and from the calibration curve *q*_v*m*_ = *f*(PCR) the volumetric flow rate value *q*_v*m*_ is read.

The causes of the deviation of the measurement points in Figure 8 during the calibrated flow meter circuit were not identified. Calibration errors in a thermal mass flow meter occur when the gas temperature or pressure deviates from the calibrated values. Calibration errors may be the result of disturbances in the output voltage, heat-transfer rate, and gas flow. The calibration accuracy is also affected by the thermophysical properties of the gas, such as thermal conductivity, diffusivity, density, specific heat, and dynamic viscosity.

## 5. Automatic Measurement of Flow Parameters

**Figure 9:**
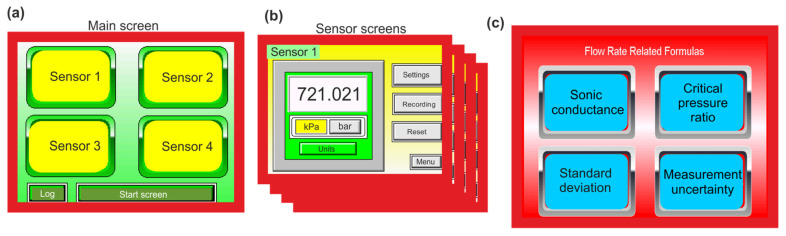
View of the main screen (**a**), and the sensor screens (**b**) of the MP panel, and screen for calculation blocks (**c**) of the CP panel.

Human–machine interface (HMI) panels were adapted as the HMI measurements panel (MP) and the control panel (CP). In MP, software was used to read and display measured values in real time, process parameter data, and perform various calculation functions. Reading the measurement values, setting threshold values (limits) for the measurement process, and downloading measurement status reports is possible using a web browser. MP provides the efficient processing of recorded measurement values, data storage, data transmission, and processing through wireless technology. MP has a built-in module for wireless communication, which is a key requirement for Industry 4.0 technology. The view of the main screen and the sensor screens of the MP panel and the screen for the calculation blocks based on the measurement parameters of the CP panel are shown in Figure 9. Four sensors are programmed on the main screen: sensor 1—upstream pressure transducer (0 to 20 MPa), sensor 2—downstream pressure transducer (0 to 20 MPa), sensor 3—upstream temperature transducer (−20 to 200 °C), sensor 4—flow meter (300 m^3^/h).

The usefulness of CP is related to the ability to control the measurement system and implement computational functions. Control algorithms were used to control pneumatic valves (proportional pressure regulator and control flow valve) during the measurement process on the measuring stand. The control algorithms for the proportional pressure regulator and the proportional flow control valve have been implemented in the CP. The proportional pressure regulator is digitally controlled in a closed-loop servo system consisting of an internal pressure transducer and an electronic valve control. The pressure output is measured using an internal pressure transducer that provides a feedback signal to the electronic valve control. The proportional flow control valve has two operating functions: in the closed position, the valve operates as a shut-off valve and in the open position, the valve operates as a proportional throttle valve. The cyclically switched on and off positions of the proportional flow control valve is controlled using pulse width modulation (PWM). The control system can be equipped with a set threshold point, which allows the arrangement of the valve operation signal when the flow rate reaches a certain value. The calculation results are displayed directly on the CP screen, saved in internal memory, and made available on the network through the built-in Web server module. Additional functions related to data processing, signal processing, state comparison, condition assessment, diagnostic reasoning, and statistical classification can be programmed. The advantage of CP is the ease of its programming, and a common database with CM allows for the quick creation of simple visualizations for the operator. It is also possible to create specialized applications based on the specific needs of the user.

## 6. Measurement Results

Measurements of the characteristics and flow parameters of a pneumatic valve were carried out, which were used on the test bench to experimentally verify the charge and discharge characteristics of a compressed air receiver tank [26]. The tested valve was the pneumatic directional spool control valve, 3/2 way, G1/4 ports, solenoid pilot, bistable, operating pressure 0 to 10 bar, nominal size 6 mm, nominal flow rate 1300 NL/min (80 Nm^3^/h) at pressure 7 bar.

**Figure 10:**
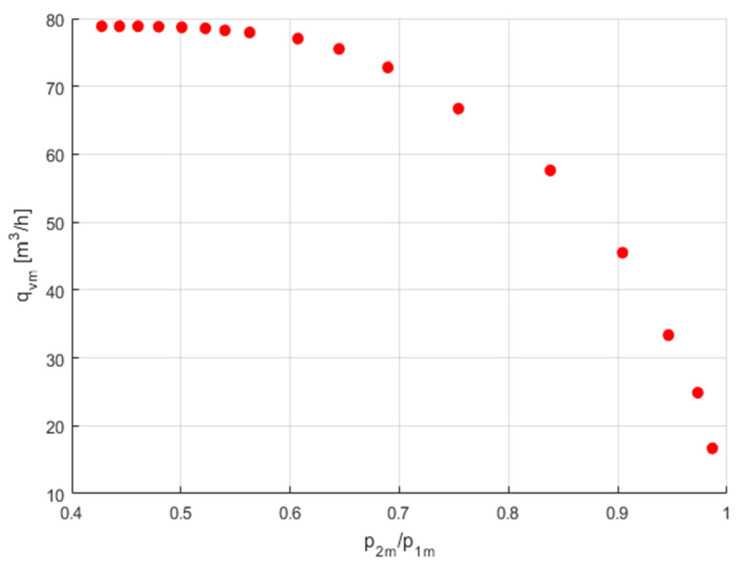
Measurement data of the flow rate.

The measurement of flow parameters *C* and *b* and the flow characteristics *q*_v*m*_ = *f*(*p*_2*m*_/*p*_1*m*_) of the pneumatic valves were carried out at a constant upstream pressure *p*_1*m*_ set by the pressure regulator and a changing downstream pressure *p*_2*m*_ adjustable through the flow control valve. The flow control valve sets the initial downstream pressure *p*_2_*_m_* below the critical point. Then, before changing the setting of this valve, the pressure drops in the pneumatic valve under test gradually increase. For each set pressure *p*_2_*_m_*, the flow rates *q*_v_*_m_* are read from the calibration curves, as seen in Figure 7 and Figure 8. The measurement data were limited to 17 points. Figure 10 shows the measurement data for the volume flow rate as a function of the pressure ratio, *q*_v*m*_ = *f*(*p*_2*m*_/*p*_1*m*_).

**Figure 11:**
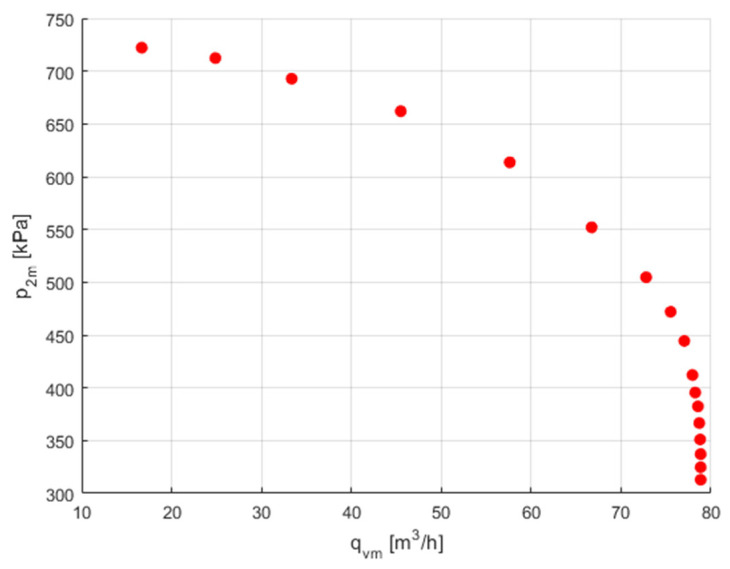
Downstream pressure measurement data.

Families of flow characteristics *p*_2*m*_ = *f*(*q*_v*m*_) obtained for different upstream pressures *p*_1*m*_ are often presented. Figure 11 shows the downstream pressure measurement data as a function of the flow ratio, *p*_2*m*_ = *f*(*q*_v*m*_).

The procedure to measure and calculate pneumatic valve flow parameters was carried out in accordance with the guidelines contained in the ISO 6358 standards and the standardized reference atmosphere given in the ISO 8778 standard [27]. The standardization of the measurement results is used to represent test results and their comparability. In pneumatic systems, the reference atmospheric conditions ANR (Atmosphere Normale de Reference) are used, with normal pressure *p_N_* = 100 kPa, temperature *T_N_* = 293.15 K, and individual gas constant *R_N_* = 288 J kg^−1^K^−1^ at 65% RH (Relative Humidity).

Based on the flow rate measurement data, the critical flow rate *q*_v_*_c_* was determined and then the volume flow sonic conductance *C* was calculated according to the formula,

(9) $$C=\frac{\frac{q_{\mathrm{vc}}}{3600}}{p_{1ma\;}\cdot 10^{3}}\;\sqrt{\frac{T_{1ma}}{T_{N}}\;}\;\frac{m^{3}}{s\;Pa}$$

Based on the flow rate measurement data, the critical pressure ratio *b* was determined, at which point the flow becomes sonic (critical)

(10) $$b=\frac{p_{2mc}}{p_{1ma}}$$

where *p*_2*mc*_ is the critical downstream pressure from the measured data.

The problem of numerically determining the critical pressure ratio *b* as a fitting parameter was solved by fitting the function in the form of a relative flow rate,

(11) $$F_{i}\left(X_{i},b\right)=\sqrt{1-{\left(\frac{X_{i}-b}{1-b}\right)}^{2}}$$

to the *i*-th measurement data of the flow rate ratio,

(12) $$Y_{i}=\frac{q_{vmi}}{q_{vmc}}$$

as a function of pressure ratio,

(13) $$X_{i}=\frac{p_{2mi}}{p_{1ma}}$$

by minimizing the least squares fit (*LSF*),

(14) $$LSF\left(b\right)=min\overset{n}{\underset{i=1}{\sum }}{\left(Y_{i}-F_{i}\left(X_{i},b\right)\right)}^{2}\;$$

**Figure 12:**
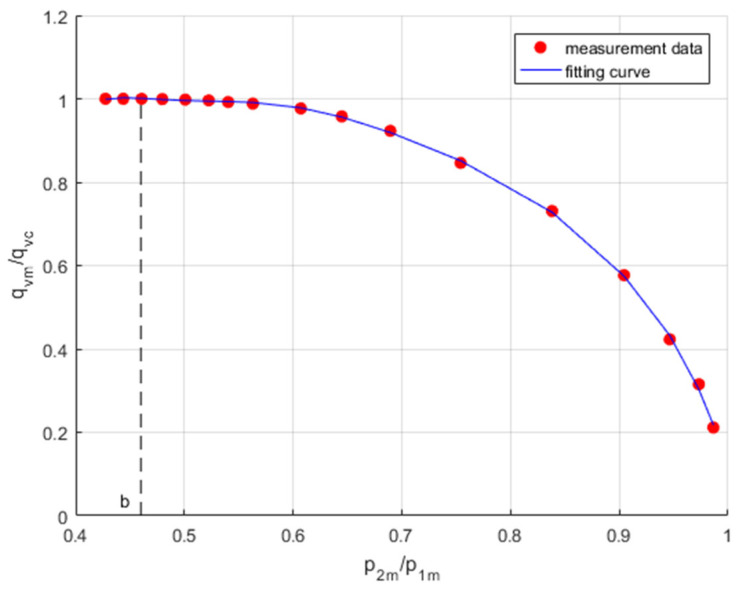
Flow rate measurement data with a fitting curve.

The application of the Matlab *lsqcurvefit* function provides a convenient interface for solving the measurement data fitting problems. The fit model uses the starting point of the critical pressure ratio *b*_0_ = 0.46, as read from the measurement data. From the *LSF* numerical procedure, the best fitting value of the critical pressure ratio *b* = 0.4781 was obtained. Figure 12 shows the flow rate measurement data with a fitting curve in the flow rate ratio vs. pressure ratio coordinates.

**Figure 13:**
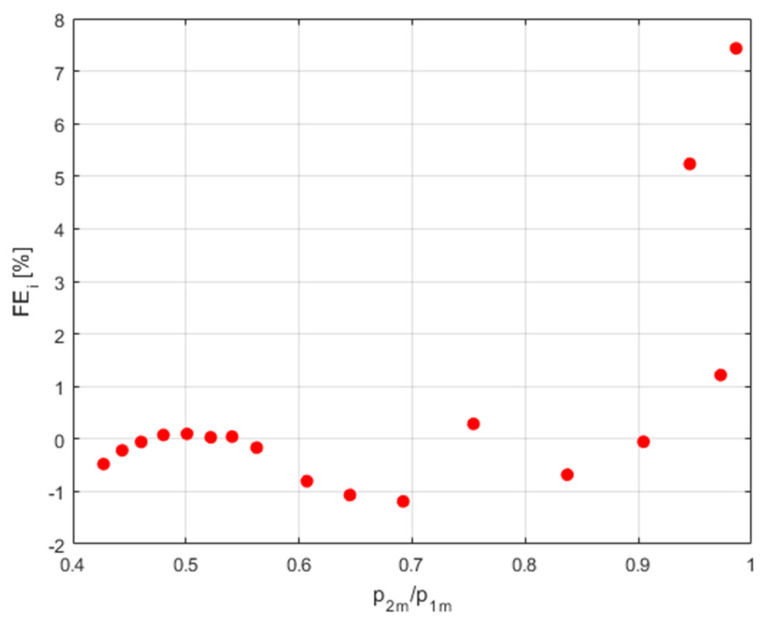
Relative fit error to the flow rate measurement data.

Figure 13 shows the relative fit error for the *F_i_*(*X_i_*,*b*) function with respect to the flow rate measurement data determined from the formula,

(15) $$FE_{i}=\frac{F_{i}\left(X_{i},b\right)-Y_{i}}{Y_{i}}\;100\%$$

Equipping the measuring station with HMI MP in CP enables an automatic measurement procedure and the evaluation of the measurement results. The standard deviation of the critical flow rate *sq*_vc_, the upstream pressure *sdp_1m_*, and the upstream temperature *sdT*_1*m*_ was calculated for a distribution of measured values. The uncertainty of the sonic conductance *sC*/*C* and the critical pressure ratio *sb*/*b* was calculated according to the ISO 6358-2 standard [28]. The results of the measurements and the values of the parameters calculated of the pneumatic valve tested are presented in Table 2.

## 7. Discussion

When the pneumatic valves were selected, the two flow ranges, choked flow and subsonic flow, were considered. The sonic conductance *C* corresponds to the constant choked flow rate. The critical pressure ratio *b* is the boundary between choked and subsonic flow. The theoretical model for calculating the volume flow rate through pneumatic valves tested under two flow conditions is as follows,

- flow rate under choked flow conditions, for $\frac{p_{2}}{p_{1ma}}\leq b$

(16) $$q_{vch}=C\;p_{1ma}\;10^{3}\sqrt{\frac{T_{N}}{T_{1ma}}\;}\;3600$$

- flow rate under subsonic flow conditions, for $\frac{p_{2}}{p_{1ma}}>b$

(17) $$q_{vsu}=q_{vch}\sqrt{1-{\left(\frac{\frac{p_{2}}{p_{1ma}}-b}{1-b}\right)}^{2}}$$

**Figure 14:**
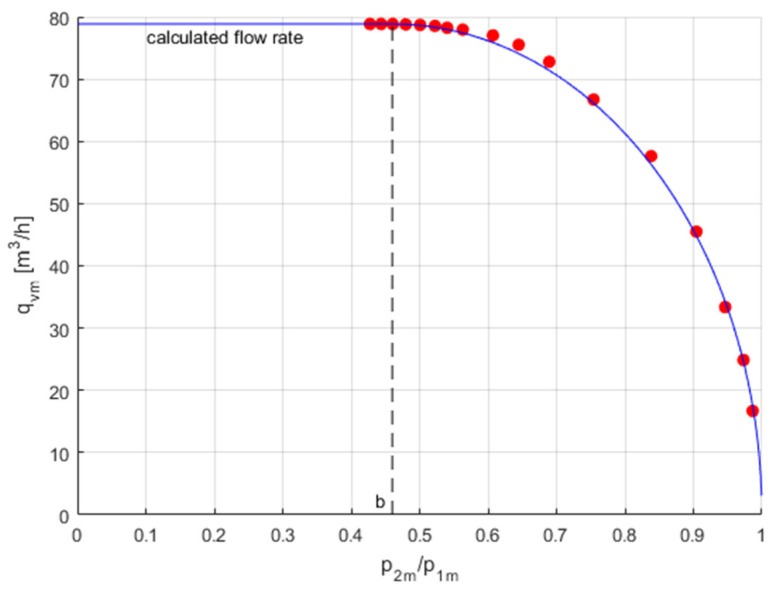
Comparison of the measurement data and the theoretical flow characteristic of the valve under test.

Figure 14 compares the measurement data and the theoretical flow rate characteristic of the valve under test.

**Figure 15:**
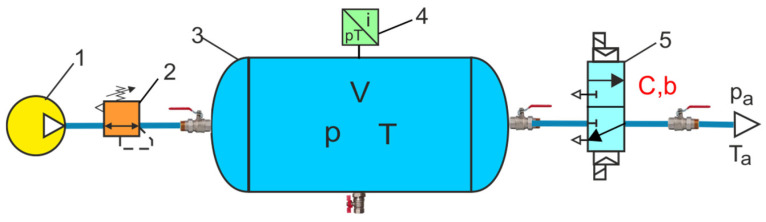
Diagram of the air tank discharge measurement test bench: 1—compressor, 2—pressure regulator, 3—stand-alone air receiver tank, 4—dual pressure and temperature transducer, 5—pneumatic solenoid valve.

The accurate determination of the flow parameters *C* and *b* of pneumatic valves is essential in evaluating the dynamic processes in air systems. This was confirmed by the discharging processes air tank tests carried out on the test bench, as shown in Figure 15.

**Figure 16:**
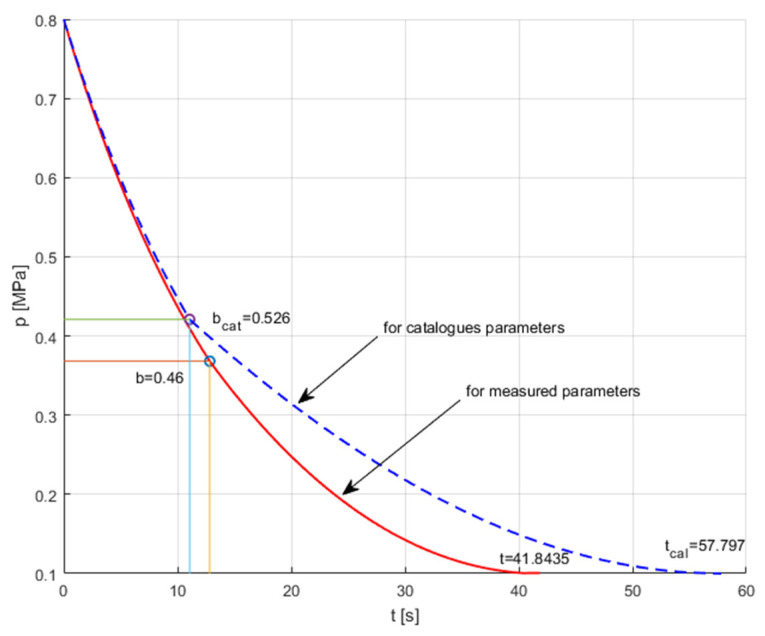
Comparison of the discharge characteristics of the air tank through the pneumatic valve for the flow parameters *C* and b, as determined on the measuring stand and adopted from the catalogue.

The discharge characteristics of the air tank were determined by assuming that the tank charged at an absolute pressure of 0.8 MPa is completely discharged at atmospheric pressure *p_a_*. Figure 16 compares the discharge characteristics of the air tank through the pneumatic solenoid valve with the flow parameters *C* and *b* determined on the measuring stand and adopted from the catalogue (*C_cat_* = 2.6 × 10^−8^ m^3^ s^−1^Pa^−1^, *b_cat_* = 0.526, for adiabatic expansion, *κ* = 1.4).

The measurement results are comparable because they were carried out in accordance with international measurement standards and can be related to ANR reference conditions,

(18) $$q_{vN}=q_{vm}\frac{\rho_{m}}{\rho_{N}}\;$$

where *ρ_m_* is the density in measurement condition and *ρ_N_* is the density referred in ANR.

## 8. Practical Purpose

**Figure 17:**
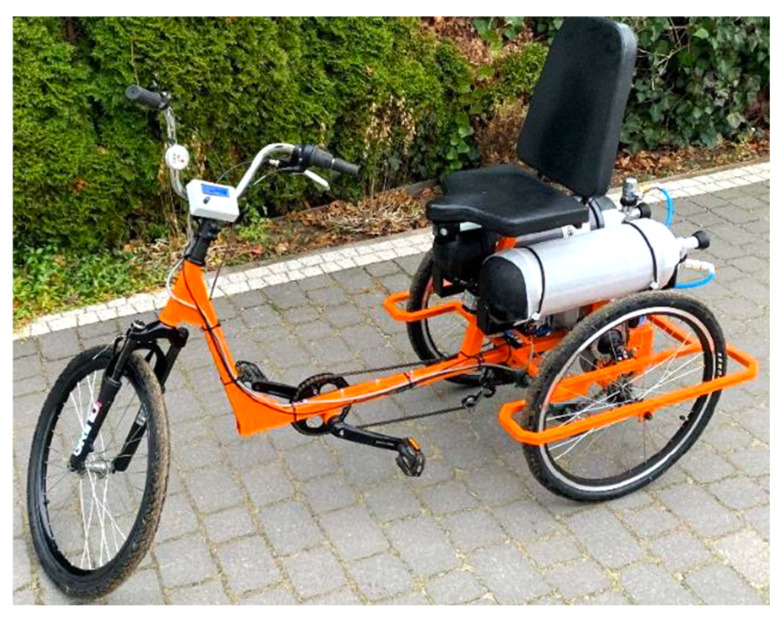
View of the HTB prototype for the rehabilitation of the elderly and disabled.

The practical purpose deals with the pneumatic propulsion system of a hybrid tricycle bike (HTB). HTB was developed as part of a research project [29] to improve the health and rehabilitation of the elderly and disabled. The HTB propulsion system is available in foot, air, and foot-to-air modes. Figure 17 shows a view of the HTB prototype for the rehabilitation of the elderly and disabled, on which the pneumatic propulsion system was experimentally tested [30].

**Figure 18:**
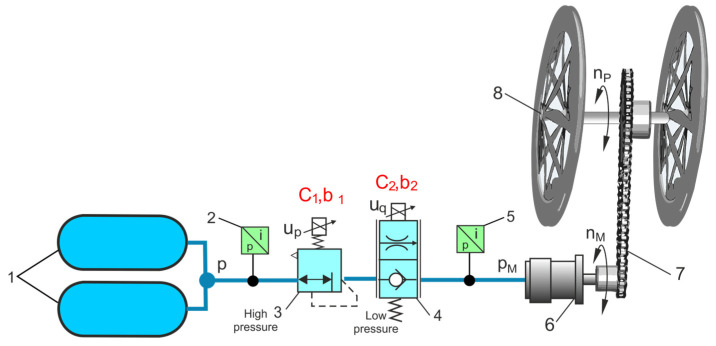
Diagram of the HTB high-pressure pneumatic propulsion system: 1—high-pressure air compressed tank, 2—pressure transducer, 3—high-pressure valve, 4—proportional throttle valve, 5—pressure transducer, 6—air motor, 7—chain transmission, 8—tricycle wheels.

Figure 18 shows a diagram of the high-pressure pneumatic drive system of the HTB in which the traction tests were performed.

**Figure 19:**
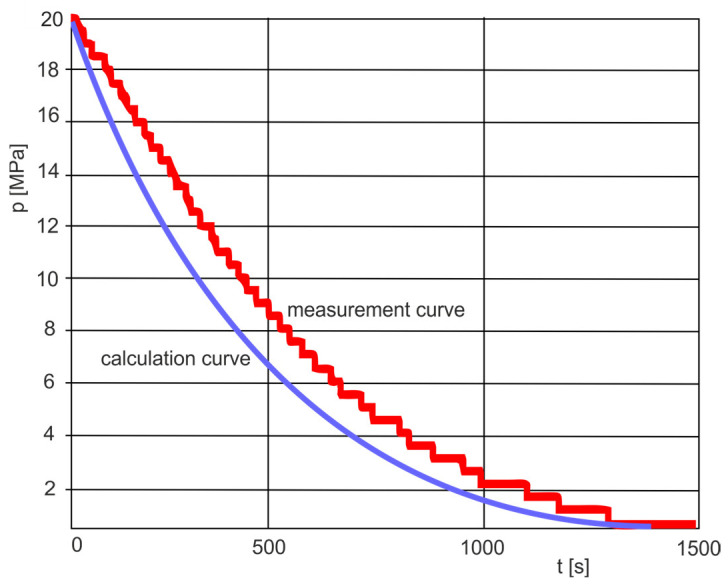
High-pressure air compressed tank discharge processes for two series connected pneumatic valves.

In the case of two valves connected in series, as shown in Figure 19, that is, a pressure valve with flow parameters *C*_1_, *b*_1_ and a proportional throttle valve with flow parameters *C*_2_, *b*_2_, the equivalent flow parameters *C*_1,2_ and *b*_1,2_ were determined. If each of the connected series valves has approximately the same capacity [31], then the values of the equivalent flow parameters *C*_1,2_, *b*_1,2_ were determined using the simplified additive method,

(19) $$C_{1,2}=\sqrt[{3}]{\frac{C_{1}^{3}\;C_{2}^{3}}{C_{1}^{3}+C_{2}^{3}}}\;$$

(20) $$b_{1,2}=1-C_{1,2}^{2}\left[\left(\frac{1-b_{1}}{C_{1}^{2}}\right)+\left(\frac{1-b_{2}}{C_{2}^{2}}\right)\right]\;$$

The numerical and experimental tests conducted consisted of determining the discharge time of the high-pressure compressed air tank using two series-connected pneumatic valves during the travel of the HTB. In the tested HTB pneumatic drive system, two composite compressed air tanks with a capacity of 2 × 9 L and a pressure of 20 MPa were used. The high-pressure valve reduces the pressure *p_C_* from 20 MPa to 0.6 MPa. The setting of the throttle valve is adapted to the air consumption and the pressure in the air motor. Figure 19 compares the numerically calculated and experimental curves of the high-pressure compressed air tanks discharge processes through two series-connected pneumatic valves. The numerical solution to discharging the compressed air receiver tank through pneumatic valves is presented in [32]. The result of the HTB high-pressure air tank discharge tests allowed us to verify the additive method of calculating the equivalent values of the flow parameters *C*_1,2_, *b*_1,2_ for two connected pneumatic valves in series. The calculation time for the tank discharging is approximately 6% shorter than the measurement time. This has no special meaning when calculating the traction time of a hybrid tricycle bike (HTB) pneumatic propulsion system, which depends on many other factors. The results presented are the basis for determining the HTB traction parameters, e.g., travel distance *S* and the selection of propulsion parameters. The speed of the HTB can be adjusted to the needs of the user by adjusting the airflow to the motor using a proportional throttle valve. An HTB pneumatic propulsion system allows for a wide range of speed settings.

The practical purpose described shows the importance of flow parameters in the series connection of pneumatic valves and their influence on the HTB’s traction. Further research will focus on determining the charging and discharging characteristics of compressed air receiver tanks (CART) through pneumatic valves of various sizes. CARTs will be used as a compressed air energy source (CAES), which is the most suitable energy storage technology for long-term and small-scale applications. The author has applied for funding for the research project “New solutions for the optimization of compressed air energy consumption and storage” from the Polish Ministry of Education and Science.

## 9. Conclusions

The main scientific contribution of this work is to solve the problem of measuring flow rates through valves with large differences in size by building a flow meter circuit, individually configured with interchangeable venturi tubes, bypass loops, and the Setaram thermal microflow meter. For the proposed measuring circuit, consisting of a parallel connection of the side loop, the main loop, and the venturi tube, the following significant relationships were formulated: the equation of the volumetric flow rate, pressure loss, the volume flow rate through the venturi tube, the volume flow rate through the heated Setaram tube, and the output voltage of the bridge. When evaluating the measurement results, a new numerical method was introduced to determine the best fit value of the critical pressure ratio *b* by fitting a function *F_i_*(*X_i_*,*b*) to the measurement data. A solution to a real scientific problem has been presented, which is to determine the discharge time of the compressed air receiver tank through series-connected pneumatic valves. The additive method for calculating the equivalent values of the flow parameters *C*_1,2_, *b*_1,2_ for two series-connected pneumatic valves was experimentally verified. The series of connecting pneumatic valves was used in the HTB high-pressure pneumatic propulsion system.

The characteristics and flow parameters of pneumatic valves of various sizes can be measured on a test bench with an individually configured flow meter circuit.

1. The proposed individually configurable flow meter circuit with the appropriate split of flow rates in the venturi/main bypass loop/side bypass loop ensures high measurement accuracy from low to large flow rates.
2. Calibration should be performed for each individually configured flow meter circuit. Measurements made on a calibrated flow meter circuit are reliable and useful in practice.
3. The automatic measurement procedure enables the precise and reproducible measurement and rapid sharing of the measurement results over a wireless network with industrial partners.
4. The theoretical model of flow parameters was well adapted to the data measured by a numeric method. The numerical model can be used to calculate the flow parameters of air valves of different sizes. Limiting the measurement points affects the precision of the fit function.
5. The discharging characteristics of the air tank through the pneumatic solenoid valve with the flow parameters *C* and *b* determined on the test bench and adopted from the catalogues were compared. The flow parameters of the valves in the catalogues do not make it possible to make accurate calculations of the discharge parameters of the compressed air tank.

## 10. Patents

Patent Number P.403007, 2015 (Poland). Compressed air delivery module. Inventors Dindorf, R. and Wos, P.

Patent Number P.426255, 2019 (Poland). Device for measuring leakage rates in pipelines for gas transmission, especially compressed air. Inventors Dindorf, R. and Wos, P.

## Figures and Tables

**Table 1:** Flow rate range for nominal sizes of venturi tubes.

Nominal Diameter *D*	Diameter Ratio *Β*	Differential Pressure Δ*p*	Flow Rate Range *q*_v_
m		Pa	m^3^/h
0.015	0.5	150	2.5
0.020	0.5	190	5
0.032	0.5	255	15
0.040	0.5	290	25
0.050	0.6	215	50
0.065	0.6	295	100
0.080	0.75	265	250

**Table 2:** Results of measurement and calculation.

Parameter	Value	Description
*p*_1*ma*_	732.014 kPa	Average upstream pressure
*sdp*_1*m*_	0.835 kPa	Standard deviation of upstream pressure
*T*_1*ma*_	295.52 K	Average upstream temperature
*sdT*_1*m*_	0.108 K	Standard deviation of upstream temperature
*q*_vc*a*_	78.85 m^3^ h^−1^	Average critical flow rate
*sq*_v*c*_	0.05 m^3^ h^−1^	Standard deviation of critical flow rate
*C*	3 × 10^−8^ m^3^ s^−1^ Pa^−1^	Volume flow sonic conductance
*sC/C*	1.8%	Uncertainty of the sonic conductance
*b*	0.4781	Critical pressure ratio
*sb/b*	2.6%	Uncertainty of the critical pressure ratio

## Data Availability

Not applicable.

## Nomenclature

*b*	critical pressure ratio
*C*	sonic conductance [m^3^/s/Pa]
*C_d_*	discharge coefficient
*D*	diameter of the throat [m]
*D*	nominal diameter [m]
*P*	pressure [Pa]
*p_a_*	atmospheric pressure [Pa]
*q_m_*	mass flow rate [m^3^/h]
*q*_v_	volumetric flow rate [kg/s]
*R*	gas constant [J/(kgK)]
*T*	temperature [K]
V	velocity [m/s]
*Β*	diameter ratio
*ε*	expansibility factor
*κ*	specific heat ratio
*ρ*	density [kg/m^3^]

## References

[1] Trujillo J.A., Gamez-Montero P.J., Codina Macia E. (2017). Air recovery assessment on high-pressure pneumatic systems. Proc. Inst. Mech. Eng. Part C J. Mech. Eng. Sci..

[2] Radgen P., Blaustein E. (2010). Compressed Air Systems in the European Union.

[3] Bhatia A. (2014). Compressors and Compressed Air Systems.

[4] Dindorf R., Wos P. (2021). Universal programmable portable measurement device for diagnostics and monitoring of hydraulic and pneumatic equipment. J. Phys. Conf. Ser..

[5] Dindorf R. (2012). Estimating potential energy savings in compressed air systems. Proc. Eng..

[6] Dindorf R., Wos P. (2021). Universal programmable portable measurement device for diagnostics and monitoring of industrial fluid power systems. Sensors.

[7] (1990). Pneumatic Fluid Power–Flow Rating Test Procedure and Reporting Method for Fixed Orifice Components.

[8] (2022). Fluidic Characteristic Quantities of Control Valves and Their Determination.

[9] (2012). Industrial Process Control Valves—Part 2-1: Flow Capacity-Sizing Equations for Fluid Flow under Installed Conditions.

[10] (2013). Pneumatic Fluid Power–Determination of Flow Rate Characteristics of Components Compressible Fluids–Part 1: General Rules and Test Methods for Steady-State Flow.

[11] Kiczkowiak T. (2009). Selection of pneumatic control valves from catalogues. J. Mech. Sci. Technol..

[12] (2016). Pneumatic Fluid Power-Determination of Flow-Rate Characteristics of Components Using Compressible Fluids–Part 1: General Rules and Test Methods for Steady-State Flow.

[13] Fojtasek K., Dvorak L., Chmura S. (2018). Experimental verification of the properties of pneumatic elements. EPJ Web Conf..

[14] Yang F., Li G., Hu D., Kagawa T. (2017). A new method for calculating the sonic conductance of airflow through a short-tube orifice. Adv. Mech. Eng..

[15] Ramsperger M., Pasieka L. (2014). The applicability of the mass-flow-model according to ISO 6358 with the parameter critical conductance C and critical pressure ratio b for gases in high-pressure range up to 300 bar. Eng. Res..

[16] Carello M., Ivanov A., Pescarmona F. (2012). Flow rate test bench: Automated and compliant to ISO standards. Exp. Tech..

[17] De las Heras S. (2001). New experimental algorithm for the evaluation of the true sonic conductance of pneumatic components using the characteristic unloading time. Int. J. Fluid Power.

[18] Dindorf R., Wos P. (2018). Automatic measurement system for determination of leakage flow rate in compressed air pipeline system. Metrol. Meas. Sys..

[19] Varga Z., Keski-Honkola P. (2012). Determination of flow rate characteristics for pneumatic valves. EPJ Web Conf..

[20] Zhang D., Gao L., Zhou S., Ma Y., Li B. (2022). Measurement of the mass-flow-rate characterization parameters of high-pressure pneumatic servo slide valves. Sci. Rep..

[21] Qian Y., GuoXiang M. (2008). Identification flow-rate characteristics of pneumatic valve by the instantaneous polytropic exponent. Meas. Sci. Technol..

[22] (1998). Guide: Mass Flow Meter for Gas and Liquid.

[23] (2003). Measurement of Fluid Flow by Means of Pressure Differential Devices Inserted in Circular Cross-Section Conduits Running Full—Part 4: Venturi Tubes.

[24] Baker R.C. (2016). Flow Measurement Handbook: Industrial Designs, Operating Principles, Performance, and Applications.

[25] (2019). Measurement of Fluid Flow in Closed Conduits–Thermal Mass Flow Meter.

[26] Dindorf R., Takosoglu J., Wos P. (2017). Development of Pneumatic Control Systems. Monograph M89.

[27] (2003). Pneumatic Fluid Power—Standard Reference Atmosphere.

[28] (2019). Pneumatic Fluid Power–Determination of Flow Rate Characteristics of Components Compressible Fluids–Part 2: Alternative Test Methods.

[29] Dindorf R., Takosoglu J., Wos P. (2021). Pneumatically assisted rehabilitation tricycle for physiotherapy of disabled patients—Design stage. Bio-Algorithms Med.-Syst..

[30] Takosoglu J., Dindorf R., Woś W., Jegier J., Sternik A., Woliński H., Marciniak J., Pusz J., Krolski J. Rehabilitation tricycle with pneumatic drive system. Proceedings of the International Scientific and Technical Conference NSHP 2023 Hydraulic and Pneumatic Drives and Controls.

[31] Rydberg K.-E. (1997). Basic Theory for Pneumatic System Design.

[32] Dindorf R., Takosoglu J., Wos P. (2023). Review of compressed air receiver tanks for improved energy efficiency of various pneumatic systems. Energies.

